# Efficacy and safety of 1565-nm non-ablative fractional laser versus long-pulsed 1064-nm Nd:YAG laser in treating enlarged facial pores

**DOI:** 10.1007/s10103-022-03622-z

**Published:** 2022-08-15

**Authors:** Ying Wang, Yuxin Zheng, Suiqing Cai

**Affiliations:** Department of Dermatology, The Second Affiliated Hospital of Zhejiang University School of Medicine, Hangzhou, Zhejiang People’s Republic of China

**Keywords:** Enlarged facial pores, Non-ablative fractional laser, Long-pulsed Nd:YAG laser

## Abstract

Facial pores are visible openings of pilosebaceous follicles, and they are one of the major factors influencing facial skin appearance. This article aims to evaluate and compare the efficacy and safety of 1565-nm non-ablative fractional laser (NAFL) and long-pulsed 1064-nm Nd:YAG laser (LPNY) in treating enlarged facial pores. All subjects were treated with NAFL on their left faces and LPNY on their right. Five treatments were administered at 2-week intervals, with one follow-up session 2 months after the final treatment. Treatment efficacy was evaluated by subjective (pore improvement and subject satisfaction ratings) assessments and objective (pore number) assessments. At each appointment, any side effects or complications were recorded to evaluate the safety of the two lasers. A total of 18 individuals participated in this study. At the 2-month follow-up, NAFL and LPNY sides had significant reduction in pores (*p* < 0.0001 and *p* < 0.0001, respectively). However, there was no statistically significant difference in the mean number of pore reductions on either side (*p* > 0.05). There was no significant difference in pore improvement ratings and satisfaction ratings between the two sides (*p* > 0.05 and *p* > 0.05, respectively). Both lasers showed minimal side effects. Both lasers effectively treated enlarged facial pores and were well tolerated. The side effects of the 1064-nm LPNY were less severe than those of the 1565-nm NAFL. ClinicalTrial.gov Identifier: NCT05360043.

## Introduction

Facial pores are visible openings of pilosebaceous follicles. These openings are not fixed structures. They can be affected by various factors, such as sebum secretion, skin elasticity, hair thickness, age, hormones, and exposure to ultraviolet radiation [1]. These enlarged pores remain a cosmetic problem impacting patients’ quality of life. Currently, there is no universal evaluation standard for enlarged facial pores, and the causes underlying enlarged facial pores remain unclear.

Various treatments have been employed to treat enlarged facial pores, with the major focus on potential causes. Treatment options include topical retinoic acid, oral isotretinoin, antiandrogen therapy, botulinum toxin type A injections, chemical peeling, lasers, radiofrequency, and ultrasound devices [2–7]. In recent years, studies on laser treatments of facial pores are gradually increasing because of good efficacy and few adverse reactions.

The long-pulsed 1064-nm Nd:YAG laser (LPNY) has been widely used for facial rejuvenation. LPNY has been demonstrated in many studies to reduce facial wrinkles and improve skin elasticity [8, 9]. It has been utilized successfully by certain researchers to treat enlarged pores [10–12]. Recently, the 1565-nm non-ablative fractional laser (NAFL) was introduced for skin resurfacing, and it has also shown promising results in treating enlarged facial pores [13]. However, there have not been enough clinical studies to confirm their efficacy, and no study has previously compared these two laser treatments. As a result, this is the first self-comparative study to compare the safety and efficacy of two lasers in the treatment of enlarged facial pores.

## Methods

### Subjects

In this study, 27 people with enlarged facial pores were enrolled and were screened by a dermatologist. Before starting the experiment, they were photographed using the VISIA Complexion Analysis System (Canfield Imaging Systems, Fairfield, NJ, USA). People would be included in the study if their pore percentile was less than 50% (i.e., their pore score is below 50% of people of the same sex, age, and skin type). Exclusion criteria included infectious skin disease or systemic disease, skin tumors, pregnancy and lactation, a history of glucocorticoids, immunosuppressant drugs and other drugs within the previous four weeks, a history of keloid, and medical history of chemical peelings, filler injections, plastic surgery, or laser therapy on the face in the last 3 months.

### Treatment protocol

All patients were treated with a 1565-nm NAFL on their left faces (ResurFX mode, M22, Lumenis®, Yokneam, Israel) for one pass, a 34–40 mJ per microbeam laser was used, and the density was 250–300 microbeam per cm^2^. Because the 1565-nm NAFL has an integrated contact cooling system, limiting bulk heating to the dermis, making it safer for darker skin. Their right faces were treated with a 1064-nm LPNY (Gentle YAG, Candela®, USA) for one pass. The spot size was 10 mm in diameter, the energy density was 45–50 J/cm^2^, the pulse width was 300 µs, and the repetition rate was 2 Hz. 1064-nm LPNY was equipped with a dynamic cooling device (DCD); the parameter was set as 40/20/0 ms. The DCD may be able to minimize skin damage by cooling the epidermis. Five treatments were carried out at 2-week intervals, with one follow-up session 2 months after the last treatment. Patients were requested to wash their faces with clean water before each treatment, and no topical lotion was applied. All patients were advised to use their own moisturizing cream for post-treatment care and avoid sun exposure.

## Evaluations

### Assessment of efficacy

Subjective and objective evaluations for improvement in the appearance of facial pores were conducted at baseline, after each treatment and 2 months after the last treatment.

The VISIA Complexion Analysis System was used to take photos of both sides of the face before each treatment and 2 months after the last treatment. The system provides a stable position and light when taking photos, reducing variability caused by camera angle and light changes. It can count the number of pores automatically. When the pore size is smaller or less visible, the system does not detect it, reducing the pore count.

Patients were asked to evaluate and rate the improvement of pore appearance after each treatment and follow-up on a 4-point grading scale shown in Table 1. In addition, patients rated their satisfaction with the treatment results using a Likert satisfaction scale (1–5) during the 2-month follow-up, as displayed in Table 2.

**Table 1:** Quartile improvement scale

0	No significant change (0–10%)
1	Mild improvement (11–25%)
2	Moderate improvement (26–50%)
3	Marked improvement (51–75%)
4	Very significant improvement (76–100%)

**Table 2:** Likert satisfaction scale

1	Very dissatisfied
2	Dissatisfied
3	Neither satisfied nor dissatisfied
4	Satisfied
5	Very satisfied

### Assessment of safety

Subjects were asked to rate the degree of pain post-treatment using a numerical rating scale (NRS) ranging from 0 (no pain) to 10 (extreme unbearable pain). At each visit, any side effects or complications, such as erythema, edema, and pigmentation, were recorded.

### Statistical analysis

Paired samples *t*-test was used to compare before and after treatment as well as the two treatment approaches. When paired *t*-test was not satisfied, Wilcoxon matched-pairs singed rank test was used to determine subjects’ assessment of the effectiveness and satisfaction score for different treatments. *p* < 0.05 was considered a statistically significant different. The software used for statistical analysis were SPSS 20.0 (IBM Corporation, Armonk, NY, USA) and GraphPad Prism 6 (GraphPad Software, San Diego, CA, USA).

## Results

Only 18 of the 27 individuals completed all treatments and were included in the final statistics. Because of poor adherence to the study protocol, nine subjects were excluded. Complaints of pain or edema, unsatisfactory treatment effects, and personal reasons accounted for the dropouts. The participants included 17 females and 1 male. All participants were aged 23 to 36 years (mean age: 27.8 years), and their Fitzpatrick skin types were III–IV.

**Fig. 1:**
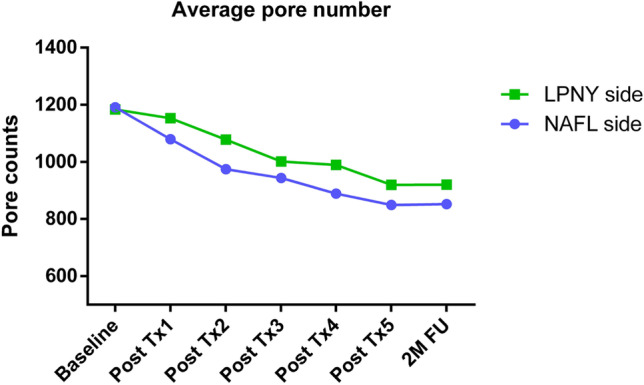
Average VISIA pore counts from baseline through 2-month follow-up visit (2 M FU). *N* = 18. Tx, treatment. NAFL, 1565-nm non-ablative fractional laser; LPNY, long-pulsed 1064-nm Nd:YAG laser

**Fig. 2:**
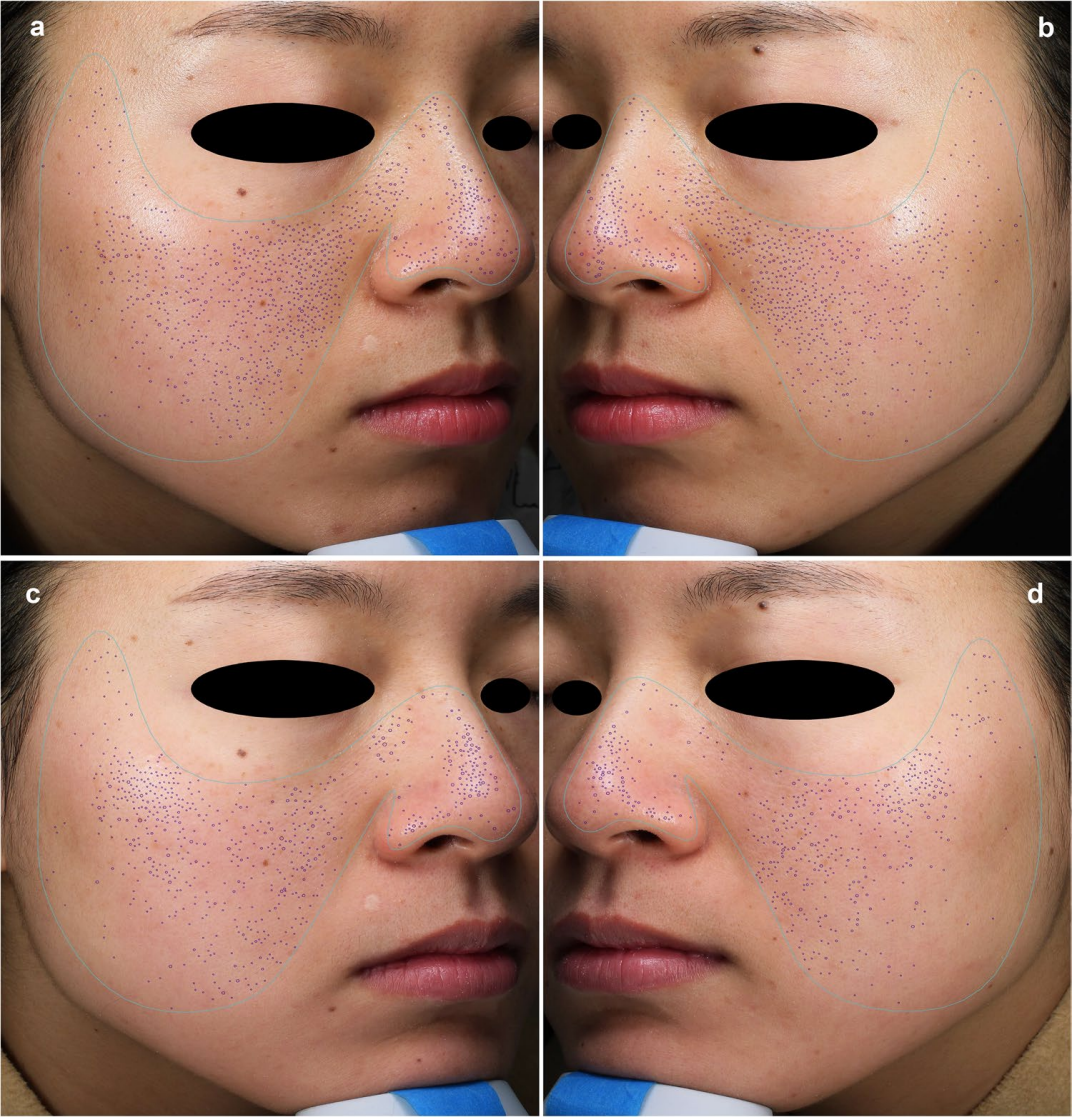
Split-face comparison of pores of a 25-year-old woman at baseline and 2-month follow-up visit with the VISIA system, highlighting the pores available for counting. **a** LPNY-treated side at baseline. **b** NAFL-treated side at baseline. **c** LPNY-treated side at 2-month follow-up, pore number decreased by 28.9% compared to baseline. **d** NAFL-treated side at 2-month follow-up, pore number decreased by 43% compared to baseline. NAFL, 1565-nm non-ablative fractional laser; LPNY, long-pulsed 1064-nm Nd:YAG laser

**Fig. 3:**
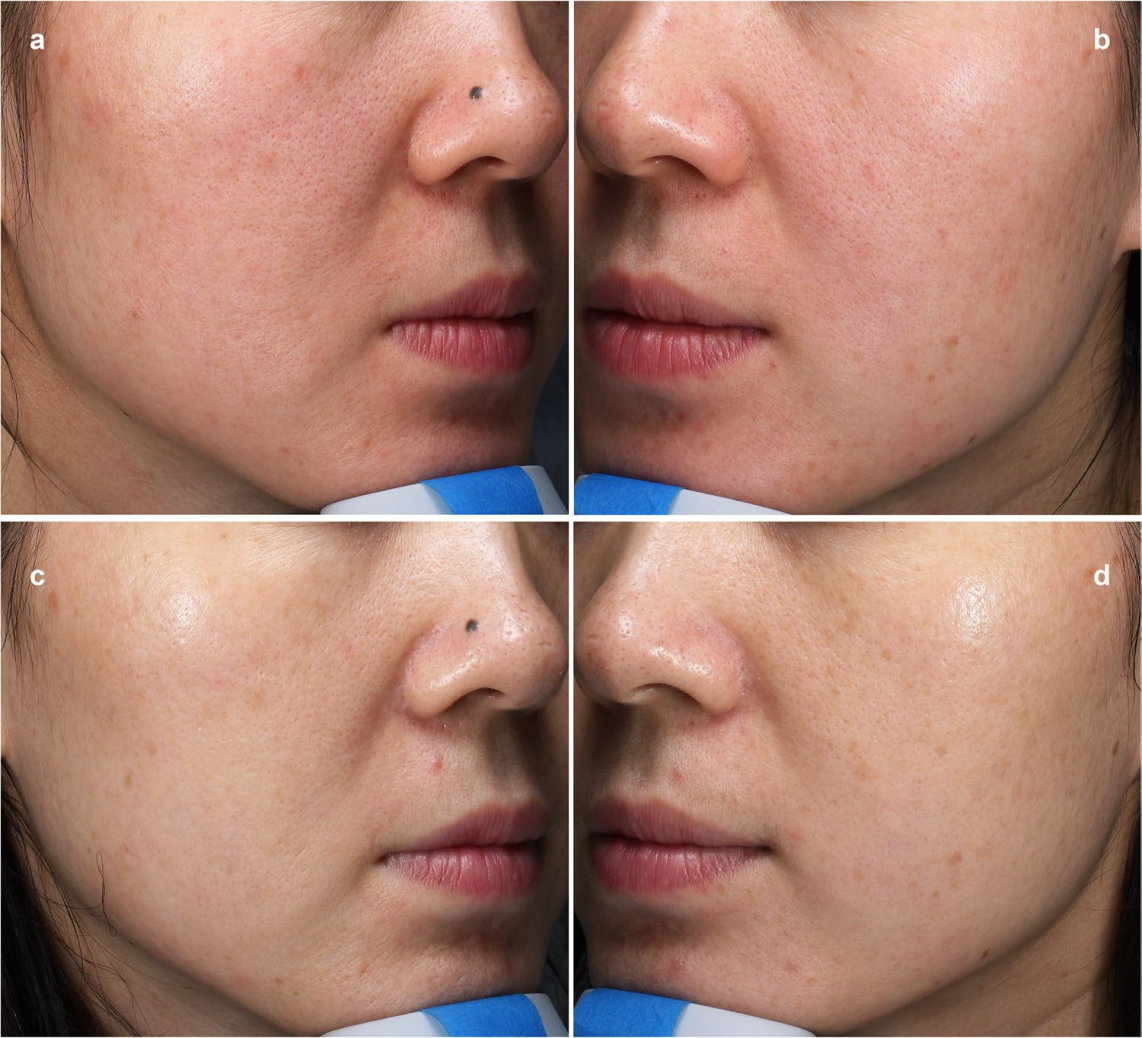
Pores of a 36-year-old woman pretreatment and at a 2-month follow-up visit after five treatments with the VISIA system. **a** LPNY-treated side at baseline. **b** NAFL-treated side at baseline. **c** LPNY-treated side at 2-month follow-up. **d** NAFL-treated side at 2-month follow-up. Visible pores were reduced after both laser treatments. More apparent improvement of pores can be observed in NAFL-treated side. NAFL, 1565-nm non-ablative fractional laser; LPNY, long-pulsed 1064-nm Nd:YAG laser

The correlation between the appearance of the number of pores detected using VISIA analysis and the time of both treatment methods are illustrated in Fig. 1. The pore count was reduced after each treatment session for both types of lasers. Before treatment, the average number of pores was 1191 ± 469.5 on the NAFL side and 1183.3 ± 520 on the LPNY side. At the 2-month follow-up, these numbers significantly reduced to 852.1 ± 372.5 and 920.5 ± 392.7 (*p* < 0.0001 and *p* < 0.0001, respectively) (Fig. 2 and Fig. 3). On the NAFL side, the average pore reduction rate was 29%, whereas, on the LPNY side, it was 21.7%. We observed that the NAFL-treated side improved significantly. However, there was no statistically significant difference in the average reduction of pores between the two sides (*p* > 0.05).

**Fig. 4:**
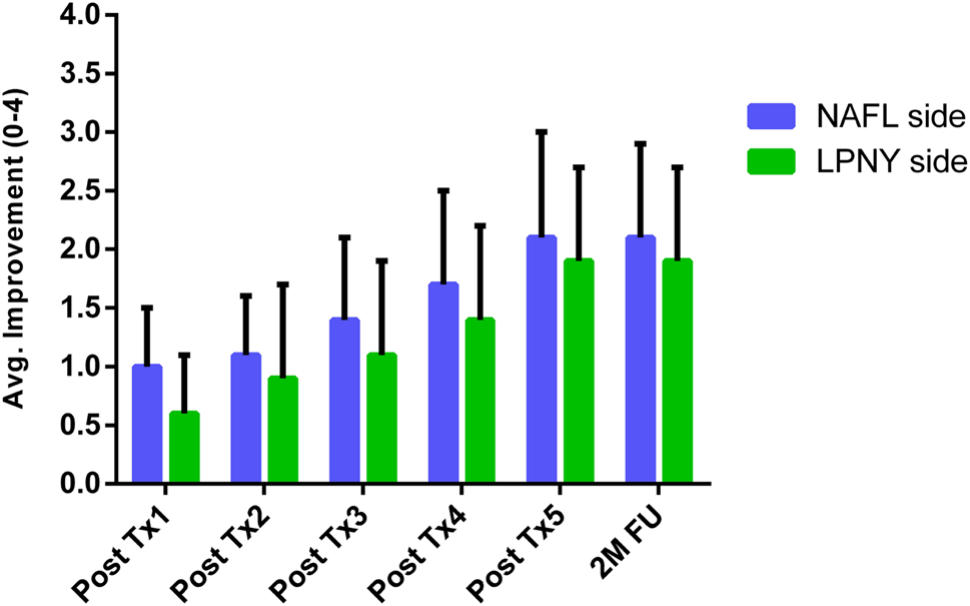
Subjective assessments. Participants rated improvement in appearance of pores after each treatment (Tx) and at 2-month follow-up (2 M FU). *N* = 18. Avg, average; NAFL, 1565-nm non-ablative fractional laser; LPNY, long-pulsed 1064-nm Nd:YAG laser

The scores of pore improvement evaluated by subjects at each visit are presented in Fig. 4. As the treatment series progressed, the scores continued to increase (Fig. 4). The average score at the 2-month follow-up was 2.1 ± 0.8 on the NAFL side and 1.9 ± 0.8 on the LPNY side, with no statistical difference between the two sides (*p* > 0.05).

In terms of satisfaction with facial pore improvement, 13 subjects (72.2%) were satisfied (score ≥ 4) on the NAFL side, and the average satisfaction score was 3.8 ± 0.9. The satisfaction rate on the LPNY side was 66.7% (12/18), with an average satisfaction score of 3.6 ± 0.9. No significant difference was observed between the two laser treatments (*p* > 0.05).

On the NAFL side, the pain was reported as mild to moderate, with an NRS score of 4.39 ± 1.24. After treatment, short-term adverse reactions included erythema and edema (100%) that persisted from a few hours to 3 days. A total of 11 subjects reported tiny scabs in the topical treatment area that lasted for 5–7 days. Temporary pigmentation was found in one individual, which completely subsided within 1 month of treatment. The pain on the LPNY side was mild, with an NRS score of 2.78 ± 1.9. The only short-term adverse reaction was mild erythema (100%). Both lasers showed no long-term adverse reactions.

## Discussion

Although numerous factors affect the number and size of pores, it is generally believed that three major factors contribute to enlarged facial pores: increased sebum secretion, reduced skin elasticity around pores, and increased hair follicle volume, especially thick hair [1]. Although many therapies have been reported, currently, there is no universally approved or effective treatment for enlarged facial pores.

This is the first self-comparative study to compare the safety and efficacy of 1064-nm LPNY and 1565-nm NAFL in the treatment of enlarged facial pores. Based on the objective evaluation of pore number by VISIA, both LPNY and NAFL induced significant therapeutic effects. Our study observed that the NAFL-treated side has more improvement, but no statistically significant difference between the two lasers was found in both objective and subjective evaluations. It could be due to the limited treatments and short follow-up time in the study.

NAFL has more noticeable side effects during and after treatment. A topical anesthetic ointment can be applied before treatment to improve treatment tolerance, and an adequate cold compress after laser treatment can shorten the duration of erythema and edema.

1064-nm LPNY is commonly used for non-ablative skin rejuvenation. According to the absorption spectra of biological pigments and water, laser energy is well absorbed by water at 1064 nm based on the correlation between the value of relative absorption and wavelength [14]. Histological studies have found that the thermal or mechanical effects of 1064-nm laser can activate fibroblasts, leading to the generation of new collagen and elastin as well as collagen remodeling [15–17]. Furthermore, the 1064-nm laser can stimulate collagen formation by inducing inflammatory responses and releasing cytokines [18, 19].

Because the 1064-nm laser has a positive effect on facial rejuvenation, it is gradually being used to treat enlarged facial pores. Wattanakrai and colleagues [12] conducted a split-face study to compare the effects of the 1064-nm LPNY with carbon Q-switched 1064-nm Nd:YAG laser followed by LPNY treatment for skin rejuvenation in Asians (20 subjects, mean age: 32.8 years). The VISIA-CR system was used in the study to count the number of pores. The number of pores on the LPNY side decreased by 32.9% compared to baseline values. They also observed that younger participants improved less from their laser treatment. Our study showed an average pore reduction rate of 21.7% on the LPNY side, which was lower than in previous studies. We assume that the difference in the results can be attributed to the difference in age groups and treatment parameters. According to other studies, the 1064-nm laser improves not only enlarged facial pores but also reduces facial sebum secretion [11, 20]. Although the exact mechanism of the 1064-nm LPNY laser’s effect on pore reduction is unknown, we can hypothesize that dermal collagen deposition and remodeling around pores may reduce the size and number of pores. The laser may also affect the sebaceous gland, reducing sebum secretion and improving pores.

The 1565-nm laser used in this study is a non-ablative fractional laser. Compared with the traditional ablative laser, such as the erbium–yttrium aluminum garnet fractional laser, NAFL causes minimal peeling on the skin surface without visible epidermal damage, minimizing adverse reactions and shortening the recovery period. Previous clinical studies have used a 1565-nm laser for the treatment of facial wrinkles, striae alba, and scars [14, 21, 22]. Yu and colleagues [13] conducted the first split-face controlled trial using a 1565-nm NAFL with objective measurements for the treatment of facial pores. The VISIA-CR system was also used to measure the number of pores. The average pore reduction rate for the NAFL side was 41.1%, which was significantly higher than the untreated side. The average pore reduction rate of our study did not reach the level of the previous study because we used lower laser intensity to prevent side effects and improve tolerance during treatments.

As 1565-nm NAFL has recently been used clinically, histological studies on its effect on the skin are limited. Previous studies have found that 1565-nm laser can promote the synthesis of types I, III, and VII collagen and elastin, as well as the remodeling of dermal collagen [14, 23]. According to previous studies, dermal collagen deposition and remodeling may be related to the mechanism by which 1565-nm laser improves facial pores.

Limitations of the present study include a limited number of participants, age groups, treatment length, no histological assessments, and lack of randomization, among others. More large-scale and long-term studies would be beneficial for further research. Furthermore, more attention should be paid to exploring the pathological mechanisms of enlarged facial pores.

## Conclusion

The 1565-nm NAFL and the 1064-nm LPNY have a similar treatment effect on enlarged facial pores, and there is no significant difference between the two lasers. The 1064-nm LPNY has fewer side effects than 1565-nm NAFL. Both lasers are well tolerated by subjects.
